# The effect of cognitive-motor dual-task training on cognitive function and plasma amyloid β peptide 42/40 ratio in healthy elderly persons: a randomized controlled trial

**DOI:** 10.1186/s12877-015-0058-4

**Published:** 2015-05-28

**Authors:** Hisayo Yokoyama, Kazunobu Okazaki, Daiki Imai, Yoshihiro Yamashina, Ryosuke Takeda, Nooshin Naghavi, Akemi Ota, Yoshikazu Hirasawa, Toshiaki Miyagawa

**Affiliations:** Osaka City University Graduate School of Medicine, 3-3-138, Sugimoto, Sumiyoshi-ku, Osaka City, Japan

**Keywords:** Attention, Amyloid peptide, Exercise, Executive function

## Abstract

**Background:**

Physical activity reduces the incidence and progression of cognitive impairment. Cognitive-motor dual-task training, which requires dividing attention between cognitive tasks and exercise, may improve various cognitive domains; therefore, we examined the effect of dual-task training on the executive functions and on plasma amyloid β peptide (Aβ) 42/40 ratio, a potent biomarker of Alzheimer’s disease, in healthy elderly people.

**Methods:**

Twenty-seven sedentary elderly people participated in a 12-week randomized, controlled trial. The subjects assigned to the dual-task training (DT) group underwent a specific cognitive-motor dual-task training, and then the clinical outcomes, including cognitive functions by the Modified Mini-Mental State (3MS) examination and the Trail-Making Test (TMT), and the plasma Aβ 42/40 ratio following the intervention were compared with those of the control single-task training (ST) group by unpaired *t*-test.

**Results:**

Among 27 participants, 25 completed the study. The total scores in the 3MS examination as well as the muscular strength of quadriceps were equally improved in both groups after the training. The specific cognitive domains, “registration & recall”, “attention”, “verbal fluency & understanding”, and “visuospatial skills” were significantly improved only in the DT group. Higher scores in “attention”, “verbal fluency & understanding”, and “similarities” were found in the DT group than in the ST group at post-intervention. The absolute changes in the total (8.5 ± 1.6 vs 2.4 ± 0.9, *p* = 0.004, 95 % confidence interval (CI) 0.75―3.39) and in the scores of “attention” (1.9 ± 0.5 vs −0.2 ± 0.4, *p* = 0.004, 95 % CI 2.25―9.98) were greater in the DT group than in the ST group. We found no changes in the TMT results in either group. Plasma Aβ 42/40 ratio decreased in both groups following the training (ST group: 0.63 ± 0.13 to 0.16 ± 0.03, *p* = 0.001; DT group: 0.60 ± 0.12 to 0.25 ± 0.06, *p* = 0.044), although the pre- and post-intervention values were not different between the groups for either measure.

**Conclusions:**

Cognitive-motor dual-task training was more beneficial than single-task training alone in improving broader domains of cognitive functions of elderly persons, and the improvement was not directly due to modulating Aβ metabolism.

## Background

Prevalence of cognitive impairment increases along with aging; for elderly persons, it causes difficulty in ADL and dependence on their family members or caregivers. Cognitive decline is also known as an evident risk factor for falling [1], which often results in being bedridden owing to hip fractures and/or other life-threatening conditions. As Lundin-Olsson et al. have long ago advised in a simple and clear manner, “Stop walking when talking” [2], elderly people with dementia are susceptible to falling when they divide their attention between two processes, which require executive cerebral functions.

Conversely, several observational studies with cognitively intact older subjects have to date revealed that high physical activity reduced the incidence of dementia [3, 4]. Furthermore, exercise intervention improves not only the essential physical elements needed to avoid falling, such as balance, muscle strength, and agility, but also cognitive functions, even in the aged individuals at high risk for dementia [5, 6]. For most of these studies, the extent of physical activity was estimated by the time spent on exercise. Moreover, in many cases, the modality of exercise was aerobic exercises, such as walking, and the details of the exercise they performed were not considered relevant. Therefore, the question of the kinds of exercises that have advantages in improving cognitive functions needs to be resolved. In particular, an evaluation of the effect of dual-task exercise training, which imposes simultaneous motor and cognitive tasks, is necessary because as indicated above, it is probable that dual-task training raises executive functions, including attention, in elderly people.

Although studies have suggested the possible mechanisms for improving cognitive function by exercise, they have not yet been fully elucidated. In pathological aspects of Alzheimer’s disease (AD), the most prevalent dementia, the deposit of senile plaques in extracellular regions, as well as intracellular neurofibrillary tangles, in the brains of the patients are observed. Amyloid β peptide (Aβ), the major component of senile plaques, is mainly composed of Aβ 40 (has 40 amino acids) and Aβ 42 (has 42 amino acids), and its clinical utility as a biomarker of AD is attracting much attention. In contrast to the levels of Aβ 40 in the cerebrospinal fluid (CSF), which are not differ depending on the stage of AD, the levels of Aβ 42 in the CSF are elevated in the early stages of AD and subsequently decrease following the progression of AD [7] owing to the deposit of senile plaques more preferentially with Aβ 42 than with Aβ 40 [8]. Recently, the possibility of low plasma Aβ 42/40 ratio as a predictor of increased risk for developing AD has also been reported [9], although the implication of plasma levels of Aβ 40 and Aβ 42 in conjunction with the variation in the levels of these proteins in CSF has not been fully elucidated. Furthermore, because Aβ peptides compete with insulin in the degradation by the common enzyme [10], it is possible that improving insulin resistance by therapeutic approach such as exercise also affects Aβ metabolism. However, it remains unclear how exercise influences the plasma Aβ 40 and 42 levels in elderly people.

For the reasons described above, we hypothesized that the intervention by dual-task training improved cognitive functions via modulating the metabolism of Aβ peptides. Therefore, in the present study, we investigated the effect of cognitive-motor dual-task training on executive cerebral functions and plasma Aβ 40 and 42 levels in healthy elderly people without marked dementia.

## Methods

### Subjects

Participants were recruited from community dwellers in Sumiyoshi-ku, Osaka City, Japan by advertising in a local magazine. The inclusion criteria were healthy, sedentary elderly people, aged over 65 years with no habit of regular exercise for more than 1 h per week. Subjects who had a history of ischemic heart disease, chronic heart failure, severe hypertension, diabetes, marked dementia with a score of under 60 for the Modified Mini-Mental State (3MS) examination, or a neuropsychiatric disorder were excluded from the study. All of these concerns were assessed using an interview sheet. The subjects who were judged by a physician to be unable or ill-equipped to participate in the exercise program were also excluded. Written informed consent was obtained from all participants, and the study protocol was approved by the ethics committee of Osaka City University Graduate School of Medicine.

### Study design

The present study was a 12-week randomized, controlled trial. It was designed as a single-blinded study, that is, the outcome assessors and the exercise instructor, but no participant knew the group identity. All applicants visited our research center in Osaka City University, and underwent baseline measurements, including the 3MS examination, an evaluation for motor ability, and laboratory analysis. The participants who met the inclusion criteria were randomly assigned to the dual-task training (DT) or the single-task training (ST) group. A disinterested third person performed the randomization by computer processing with the random number generation program. In brief, the person gave the different random number to each participant on the computer software, and ranked them due to the random number. The participants who had the ranking of even number were assigned to the DT group, and odd number to the ST group. The above operation was repeated until the person achieved balance on gender, age, years of education, and the total scores of 3MS exam. Subsequently, the subjects in each group engaged in the 12-week exercise program provided at Sumiyoshi Sports Center, a gymnasium located in Osaka City. After the intervention, all the clinical parameters were re-examined.

### Exercise intervention

All participants in the DT and the ST groups received 1-hour exercise training separately (in separate rooms for each group), three times a week, for 12 weeks. All sessions were supervised by a trained instructor.

A training session comprised 15 min of mental gymnastics mainly made up of complicated motion of the fingers, 25 min of resistance training, 10 min of aerobic exercise, and finally 10 min of systemic flexibility exercise composed by 8―10 poses. Resistance training gradually progressed from exercises like knee extensions or thigh-raises in a sitting position to squats or back-kicks in a standing position, or push-ups, or hip-raises in a recumbent position on a mat. Aerobic exercise included stepping, simple walking, and zigzag walking with cones.

In the sessions for the DT group, concurrent cognitive tasks were performed during resistance training and aerobic exercise. For instance, arithmetic tasks (subtraction of one digit) or Shiritori, a Japanese word chain game in which one player has to say a word starting with the last character of the word given by the previous player, was carried out during thigh-raises. Otherwise, the subjects switched direction, walking either forward or backward, according to the patterns of whistling. On the other hand, the subjects in the ST group received simple resistance and aerobic training.

### The Modified Mini-Mental State (3MS) examination

Cognitive assessment for all subjects was conducted using the Japanese version of the 3MS examination, which was faithfully translated from the original developed by Teng [11]. The 3MS exam comprises 15 questions, which are categorized into 8 domains, i.e., “registration & recall” (immediately and delayed), “long-term memory” (date and place of birth), “orientation” (temporal and spatial), “attention” (mental reversal), “verbal fluency & understanding” (naming, repetition and writing a sentence, reading and obeying an order, three-stage command), “word retrieval” (four-legged animals), “visuospatial skills” (copying two pentagons), and “similarities” (a point in common between two words). We graded each domain, as well as the total scores (full marks = 100), of the 3MS exam.

### Trail-Making Test (TMT)

The Trail-Making Test (TMT) was performed for the purpose of evaluating visual information processing speed. In general, the TMT comprises asking a subject to draw a line connecting 25 consecutive circled numbers sequentially as quickly as possible. We enabled easier and more systematic test processing by using a dedicated device for TMT with a touch panel (Nounenreikei ATMT, elk Corp, Tokyo, Japan), in which a subject could pick the targeted circled number by touching it instead of drawing a line. In the setting of the device, circled numbers were laid out fixed on the liquid crystal display screen, and the touched number was immediately deleted and a new one added each time a subject touched a number. The time taken in seconds to complete all 25 numbers was calculated automatically and used as the result.

### Physiological performance

We assessed isometric muscle strength of quadriceps using the strain gauge dynamometer (ST-200S, MULTECH, Japan). The subjects first sat on a chair with their hips and knees flexed at 90° and their thighs fixed to the chair using the seat belt. Then they wore the strap attached to the dynamometer round their ankles and tried to exert maximal isometric knee extension. They performed two trials in each leg and the maximum value was adopted.

To quantify motor ability, the functional tests were performed; maximal step length (MSL), the Timed Up & Go (TUG) test, and single leg standing. MSL was measured as the maximum possible length of the subject’s stride of one step. In the TUG test, we measured the time required for a subject to stand up from a chair, walk a distance of 3 m, turn, walk back to the chair, and sit down. We also measured the maximum time a subject could stand on one leg. In cases where a subject was able to continue single-leg standing for over 120 s, the test was completed at that point. These functional tests were each performed twice, and the best values were adopted.

### Anthropometry

Body mass index (BMI) was calculated as body weight × (height)^−2^ and expressed in kilograms per square meter. Percentages of body fat and muscle mass of lower extremities were estimated by bioelectrical impedance analysis using the body composition analyzer (Nippon Shooter Ltd., Physion MD, Tokyo, Japan).

### Laboratory measurements and evaluation of insulin sensitivity

Blood samples were collected after a 12-hour overnight fast. Plasma glucose levels were measured by the hexokinase UV method and serum insulin levels by chemiluminescent enzyme immunoassay. We calculated the homeostasis model assessment of insulin resistance (HOMA-IR), an established surrogate index of insulin resistance [12]. The HOMA-IR was obtained from fasting plasma glucose (FPG) and serum insulin (FIRI) levels according to the original method by Matthews et al. [13], with the following formula:

HOMA-IR = FPG in mg/dl × FIRI in μU/ml/405

A greater HOMA-IR represents a higher insulin resistance.

Plasma Aβ 40 and 42 levels were measured by a commercially available enzyme-linked immunosorbent assay kit (#298-64601 for Aβ 40 and #296-64401 for Aβ 42, Wako, Osaka, Japan). Regarding the test performance of the kit, the sensitivity was 0.019 pmol/l (dynamic range, 1.0–100 pmol/l) for Aβ 40 and 0.06 pmol/l (dynamic range, 0.1–20 pmol/l) for Aβ 42. The inter- and intraassay coefficients of variation were less than 10 %.

### Statistical analysis

Comparisons of mean values at baseline and after the intervention between groups were performed by unpaired *t*-test. The changes in clinical parameters following intervention in each group were examined by paired *t*-test. Comparisons of the absolute changes in scores in the 3MS exam and each of its domains, following intervention, were also performed by unpaired *t*-test. All statistical procedures were performed using SPSS (IBM, NY, US) for Windows (Microsoft Inc. WA, US). P values less than 0.05 were considered statistically significant. Ninety-five percent of confidence intervals (95 % CI) were calculated to estimate the strength of the association when the p value for the group comparison was significant.

## Results

### Clinical characteristics of the subjects

**Fig. 1:**
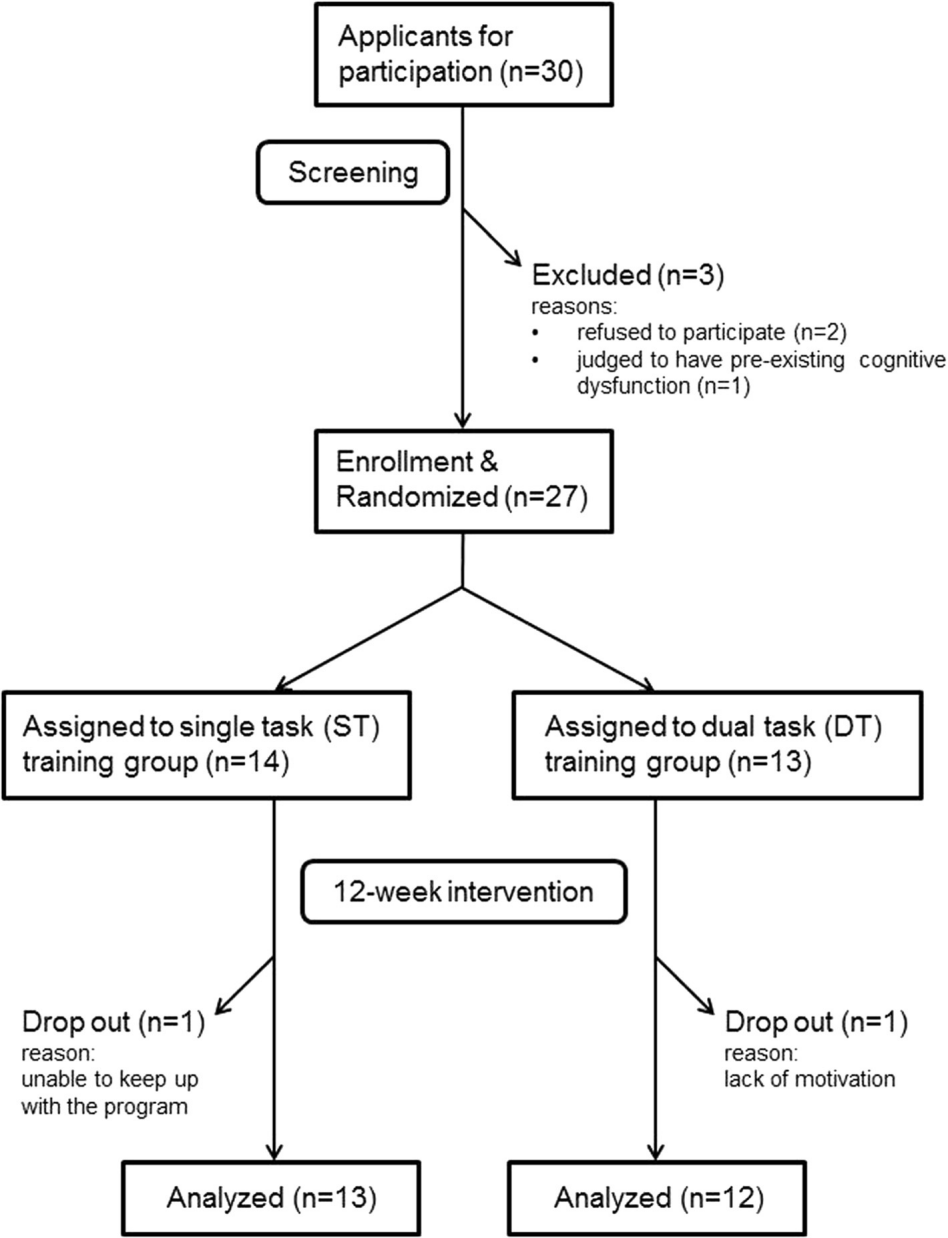
Flow chart on the study design

Participants were recruited between February and March 2014. The flow chart of screening, baseline measurement, enrollment, randomization, intervention, and data analysis is shown in Fig. 1. There were 30 applicants in the present study. Two of them were excluded from the study because of their refusal to participate, having given their written consent. Another applicant who scored only 49 in 3MS was also excluded. The remaining 27 applicants were assigned to either the ST (n = 14) or the DT (n = 13) group. One participant from each group dropped out during the intervention period.

Mean adherence to the intervention was 96.5 % in the ST group and 90.2 % in the DT group. We confirmed that all 25 subjects were engaged in more than 80 % of all sessions.

The clinical characteristics of the subjects are shown in Table 1. Mean ages were 74.2 ± 3.4 years and 74.2 ± 4.3 years in the ST and DT groups, respectively. Mean years of education were 12.0 ± 1.8 years and 11.9 ± 1.7 years in the ST and DT groups, respectively. At baseline, there were no differences in body composition, muscular strength, and motor ability between the groups.

**Table 1:** Clinical characteristics, body composition, muscular strength, and motor ability, before and after a 12-week intervention

		ST group		DT group	
		Baseline	Post	Baseline	Post
Gender	(M/F)	1/12	---	1/11	---
Age	(years)	74.2 ± 3.4	---	74.2 ± 4.3	---
Education	(years)	12.0 ± 1.8	---	11.9 ± 1.7	---
SBP	(mmHg)	144 ± 15	137 ± 22	138 ± 18	140 ± 16
DBP	(mmHg)	82 ± 7	82 ± 11	84 ± 12	79 ± 7
BMI	(kg/m^2^)	21.7 ± 2.9	21.3 ± 3.0*	24.2 ± 4.5	23.8 ± 4.2*
Body fat	(%)	26.4 ± 6.6	25.3 ± 5.8	30.5 ± 6.6	30.2 ± 5.4**
Leg muscle mass	(kg)	8.2 ± 1.6	7.7 ± 1.5*	9.1 ± 2.2	9.0 ± 1.9
Quad. muscle strength	(kg)	24.1 ± 6.6	28.5 ± 6.2*	24.9 ± 6.5	28.8 ± 7.4*
MSL	(cm)	97.3 ± 14.6	107.7 ± 12.1*	94.4 ± 15.8	100.0 ± 16.1*
TUG	(sec)	5.82 ± 1.38	5.54 ± 1.00	5.84 ± 1.31	5.93 ± 1.10
Single-leg standing	(sec)	71.7 ± 48.3	83.3 ± 46.8	68.2 ± 43.3	78.8 ± 42.4

All values are presented as n or mean ± SD

Abbreviations: *M* male, *F* female, *SBP* systolic blood pressure, *DBP* diastolic blood pressure, *BMI* body mass index, *MSL* maximal step length, *TUG* Timed Up & Go test

*p < 0.05 within the group

**p < 0.05 between the groups

### Effects on muscle mass and motor ability

The changes in body composition, muscle strength, and motor ability following the 12-week training in both groups are shown in Table 1.

Muscular strength of quadriceps was equally improved following the training in both groups (ST group: 24.1 ± 6.6 to 28.5 ± 6.2 kg, *p* = 0.002; DT group: 24.9 ± 6.5 to 28.8 ± 7.4 kg, *p* = 0.022), and there was no difference between the groups at post-intervention. With regard to motor ability, MSL was improved in both groups (ST group: 97.3 ± 14.6 to 107.7 ± 12.1 cm, *p* = 0.006; DT group: 94.4 ± 15.8 to 100.0 ± 16.1 cm, *p* = 0.014), with no significant difference at 12 weeks. No significant changes in other parameters of motor ability were found following the training in either group.

### The outcome in cognitive function

The results of the 3MS exam and the TMT before and after the intervention in both groups are shown in Table 2. At baseline, there were no differences in the total scores of the 3MS exam and the scores of each of its domains of cognitive function, as well as in the results of the TMT.

**Table 2:** The results of the Modified Mini-Mental State (3MS) examination and the Trail-Making Test (TMT)

		ST group		DT group	
	(Full marks)	Baseline	Post	Baseline	Post
3MS total scores	(100)	90.6 ± 2.2	93.0 ± 2.5*	89.3 ± 1.7	97.8 ± 0.5*
Registration & recall	(21)	20.0 ± 0.5	20.7 ± 0.2	18.8 ± 0.8	20.6 ± 0.3*
Long-term memory	(5)	5.0 ± 0.0	4.8 ± 0.2	4.9 ± 0.1	5.0 ± 0.0
Temporal & spatial orientation	(20)	18.8 ± 0.9	18.5 ± 1.1	18.4 ± 0.8	19.8 ± 0.3
Attention	(7)	5.7 ± 0.4	5.5 ± 0.5	5.1 ± 0.5	7.0 ± 0.0*^,^**
Verbal fluency & understanding	(21)	20.5 ± 0.1	20.4 ± 0.2	20.3 ± 0.3	21.0 ± 0.0*^,^**
Word retrieval	(10)	8.3 ± 0.6	9.5 ± 0.3*	9.6 ± 0.2	9.8 ± 0.1
Visuospatial skills	(10)	9.9 ± 0.1	9.7 ± 0.3	9.7 ± 0.1	10.0 ± 0.0*
Similarities	(6)	2.5 ± 0.5	3.5 ± 0.4*	2.6 ± 0.4	4.7 ± 0.3*^,^**
TMT (seconds)	---	69.6 ± 10.9	78.0 ± 15.4	70.2 ± 4.8	71.0 ± 3.7

All values are presented as mean ± SE

*p < 0.05 within the group

**p < 0.05 between the groups

The total scores in the 3MS exam were improved following the training, in both groups (ST group: 90.6 ± 2.2 (SE) to 93.0 ± 2.5, *p* = 0.023; DT group: 89.3 ± 1.7 to 97.8 ± 0.5, *p* < 0.001), but the scores at post-intervention were not different between the groups. Regarding the scores for each domain in the 3MS exam, there were significant improvements in “registration & recall” (18.8 ± 0.8 to 20.6 ± 0.3, *p* = 0.035), “attention” (5.1 ± 0.5 to 7.0 ± 0.0, *p* = 0.003), “verbal fluency & understanding” (20.3 ± 0.3 to 21.0 ± 0.0, *p* = 0.043), and “visuospatial skills” (9.7 ± 0.1 to 10.0 ± 0.0, *p* = 0.039) only in the DT group. The scores in “attention”, “verbal fluency & understanding”, and “similarities” were significantly higher in the DT group than in the ST group at post-intervention (7.0 ± 0.0 vs 5.5 ± 0.5, *p* = 0.008, 95 % CI 0.46―2.47; 21.0 ± 0.0 vs 20.4 ± 0.2, *p* = 0.040, 95 % CI 0.02―0.60; 4.7 ± 0.3 vs 3.5 ± 0.4, *p* = 0.038, 95 % CI 0.08―2.18, respectively).

**Fig. 2:**
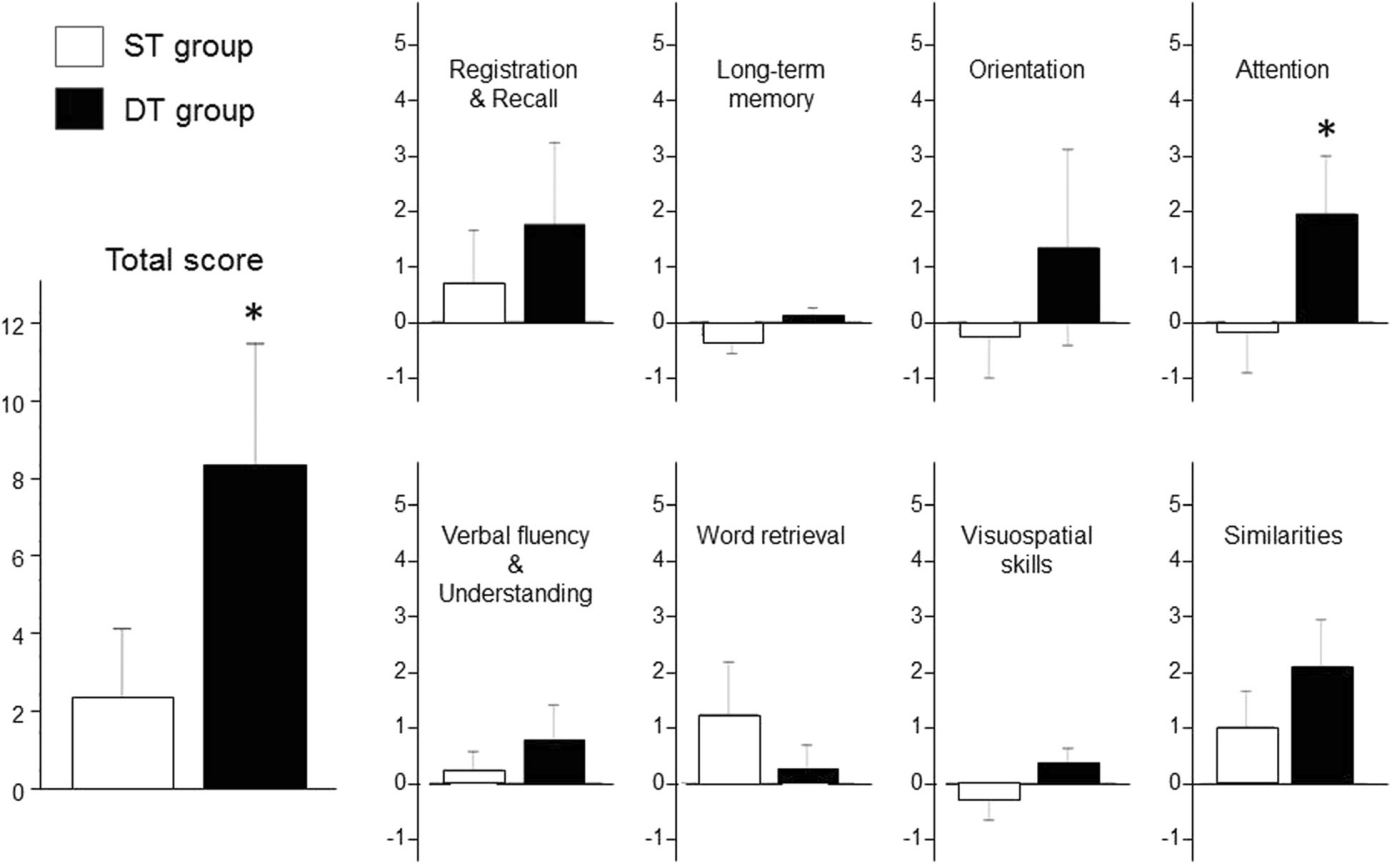
The absolute changes in the scores of the 3MS exam following the 12-week intervention in both groups. The absolute changes in the scores of “attention” (1.9 ± 0.5 vs −0.2 ± 0.4, p = 0.004, 95 % CI 0.75―3.39) as well as in the total score (8.5 ± 1.6 vs 2.4 ± 0.9, *p* = 0.004, 95 % CI 2.25―9.98) were greater in the DT group than in the ST group. The horizontal bars represent means ± SE. *: *P* < 0.01

Then, we compared the absolute changes in the total scores and in each domain of the 3MS exam, following the training, between the groups. The results are shown in Fig. 2. The absolute changes in the scores of “attention” (1.9 ± 0.5 vs −0.2 ± 0.4, *p* = 0.004, 95 % CI 0.75―3.39), as well as in the total score (8.5 ± 1.6 vs 2.4 ± 0.9, *p* = 0.004, 95 % CI 2.25―9.98) were greater in the DT group than in the ST group.

We found no improvements in the results of TMT in either group.

### Effects on plasma Aβ 42 and Aβ 40 levels and Aβ 42/40 ratio associated with insulin sensitivity

At baseline, no differences were found in plasma Aβ 42 and Aβ 40 levels or the Aβ 42/40 ratio between the groups. Plasma Aβ 42 levels were decreased (ST group: 8.0 ± 1.3 (SE) to 3.0 ± 0.6 pmol/l, *p* = 0.003; DT group: 8.0 ± 1.5 to 5.0 ± 1.1 pmol/l, *p* = 0.170) and Aβ 40 levels were increased (ST group: 14.4 ± 1.1 to 20.5 ± 1.8 pmol/l, *p* = 0.007; DT group: 14.5 ± 0.9 to 20.5 ± 1.9 pmol/l, *p* = 0.002) following the intervention in both groups, but the change in Aβ 42 was not statistically significant in the DT group.

**Fig. 3:**
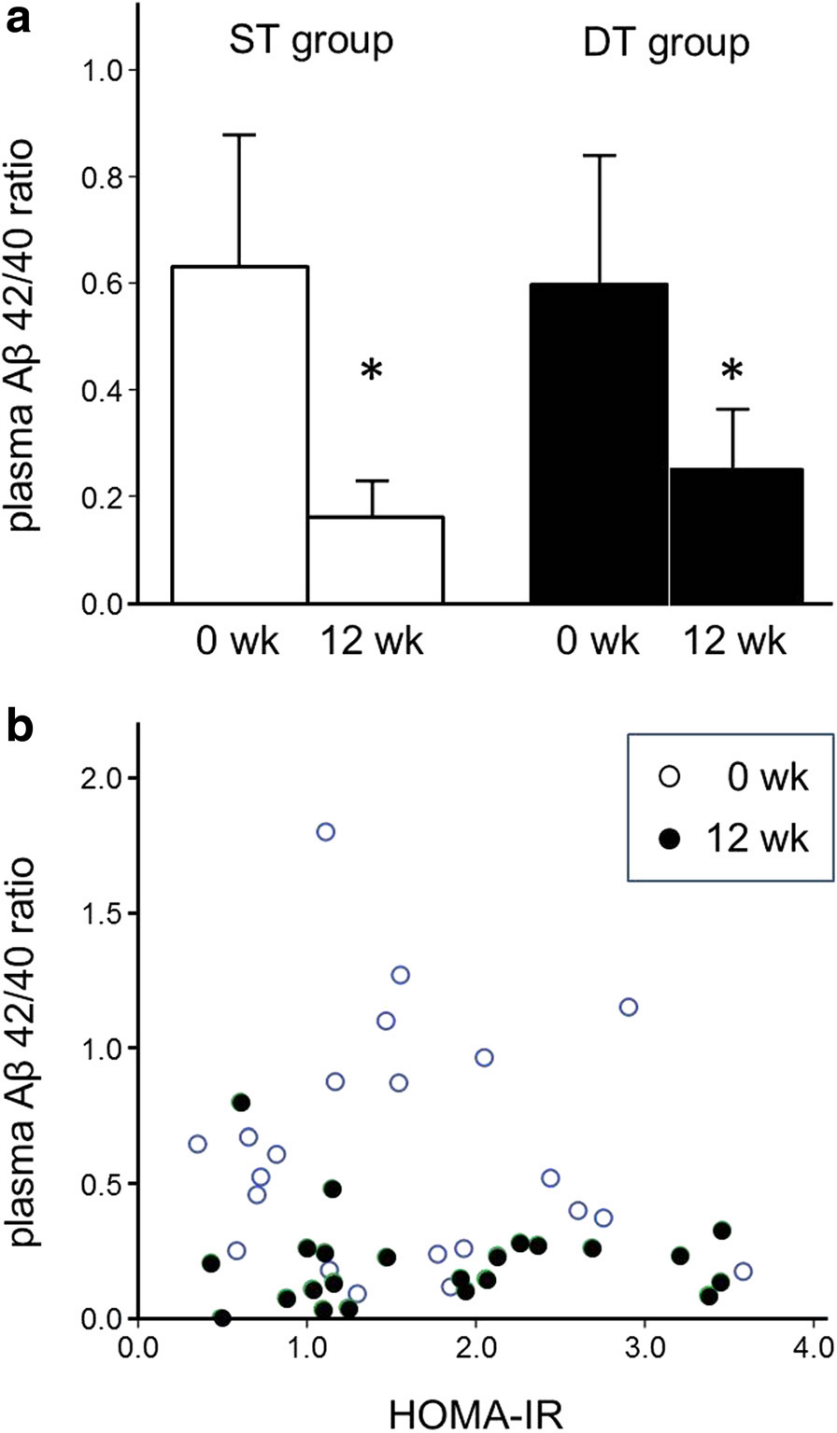
**a** Plasma amyloid β peptides (Aβ) 42/40 ratio before and after the intervention. Significant decreases in Aβ 42/40 ratio were found in both groups following the training (ST group: 0.63 ± 0.13 to 0.16 ± 0.03, *p* = 0.001; DT group: 0.60 ± 0.12 to 0.25 ± 0.06, *p* = 0.044), although the post-intervention value was not different between the groups. **b** The correlation between the HOMA-IR and the plasma amyloid β peptides (Aβ) 42/40 ratio. In all subjects, there was no significant correlation between the HOMA-IR and the Aβ 42/40 ratio in both pre- and post-intervention states

As shown in Fig. 3a, significant decreases in the Aβ 42/40 ratio were found in both groups following the training (ST group: 0.63 ± 0.13 to 0.16 ± 0.03, *p* = 0.001; DT group: 0.60 ± 0.12 to 0.25 ± 0.06, *p* = 0.044), although the post-intervention values were not different between the groups. We could not find any changes in the HOMA-IR following the intervention in either group (ST group: 1.22 ± 0.66 to 1.64 ± 1.02 pmol/l, *p* = 0.078; DT group: 2.03 ± 0.90 to 1.90 ± 0.94 pmol/l, *p* = 0.843). In all subjects, there was no correlation between the HOMA-IR and the Aβ 42/40 ratio, both at pre- and at post-intervention (Fig. 3b).

## Discussion

The objective of the present study was to investigate the impact of cognitive-motor dual-task training on executive cerebral functions and plasma Aβ levels in cognitively independent healthy elderly people. We were able to show that broader domains of executive functions, including attention, were improved by the cognitive-motor dual-task training, than by its single-task counterpart. On the other hand, plasma Aβ 42/40 ratios were significantly decreased following the intervention, regardless of the type of exercise. These results suggest that cognitive-motor dual-task training is more beneficial than single-task training alone in improving cognitive function in elderly people and that its superiority is not directly due to the modulation of the Aβ metabolism.

Not only preserving physical strength but also remaining cognitively intact is a crucial factor in preserving self-reliance in ADL for elderly people. Physical activity is an important factor in reducing the incidence of dementia [3, 4] as well as other lifestyle-related diseases, such as hypertension, diabetes, and cardiovascular disease. Furthermore, previous clinical interventions revealed the favorable influence of exercise on cognitive function evaluated by grading scales, such as the 3MS or Mini-Mental State exam [14], and on brain morphology, especially in hippocampal volume [15]. Thus evidence is accumulating to establish exercise as a non-pharmacological therapeutic strategy to counter cognitive decline. The advantage of dual-task training demonstrated in our results could add inventiveness to the construction and development of specific exercise programs targeting the improvement of cognitive function.

To date, studies have suggested the mechanism of improving cognitive function by exercise. Increase in cerebral blood flow [16] and angiogenesis [17], as well as anti-oxidant action [18], are generally considered to be the candidates. However, these effects, resulting mainly from aerobic exercise in most previous reports do not seem to explain the superiority of dual-task training on specific cognitive domains because both types of training in the present study equally included the aspect of aerobic exercise in each session. Recently, various cellular and molecular mechanisms have been elucidated by which physical activity enhances neuroplasticity [19] and the interconnection [20] associated with increasing neurotrophins, in particular, brain-derived neurotrophic factor (BDNF). The superior effects of dual-task training on executive functions may be explained by these exercise-induced changes in the brain regions, although it remains to be further investigated whether these changes could be modified when motor training is performed concurrently with cognitive tasks.

Focusing on each cognitive domain, many researchers have indicated that the positive effect of exercise was apparent on attention, the most fundamental faculty of all executive functions [21, 22]. Our results, which showed a greater improvement in attention in the DT group, were consistent with previous findings. The improvement of verbal fluency found only in the DT group in the present study also supports the findings in the recent systematic review by Cooper et al., in which they concluded that this function was improved by group exercise programs [23]. The regions of the hippocampus involved in performing various tasks are different according to each cognitive domain [24]; furthermore, as reported by Rosano et al. in their cross-sectional study using functional MRI, there are heterogeneity in regions of the brain activated by physical activity [25]. Although these phenomena may explain in part the diversity in the improvement of cognitive domains by exercise, we believe that the construction of the exercise program in the present study, in which many tasks including the switching of attention or linguistic representation were included, resulted in the improvement in the specific domains of executive function in our results. From this viewpoint, there remains room for improvement in developing the program for the enhancement of visual information processing speed, an attribute we also failed to improve, as assessed by the TMT, as several previous studies have reported.

To the best of our knowledge, this is the first report investigating the effect of exercise on plasma Aβ levels in humans. We expected dual-task training could delay the spontaneous decreasing in plasma Aβ 42/40 ratio compared with single-task training. However, our 12-week exercise intervention brought about significant decreases in the plasma Aβ 42/40 ratio equally in both groups, a result that countered our expectations. This result suggests that the improvements in the broader domains of executive functions by DT training, rather than by ST training, are not directly due to the modulation of the Aβ metabolism. It is well known that Aβ peptides are metabolized by the insulin-degrading enzyme (IDE) that also metabolize insulin [10] and that hyperinsulinemia due to peripheral insulin resistance and conditions associated with impaired glucose metabolism, such as obesity, or type 2 diabetes, are linked to cognitive dysfunction and AD [26]. Furthermore, acute insulin infusion increases plasma concentrations of Aβ peptides [27]. Therefore, we also examined the correlation between the HOMA-IR, the surrogate index of insulin resistance, and the plasma Aβ 42/40 ratio, but we could not find any significant correlation between these factors or any changes in the HOMA-IR (at least following the 12-week intervention) in either group. The lack of correlation between insulin resistance and Aβ peptides in our study supports the findings by Baker et al., in which they showed improvement in the insulin sensitivity of elderly people with glucose intolerance, following 6 months of aerobic exercise, with no significant changes in plasma levels of Aβ peptides [28]. The reduction in BMI associated, in part, with the reduction in body fat in the present study and increased fat oxidation generally accompanied by resistance training may be an explanation for the decreased plasma Aβ 42/40 ratio, regardless of the groups, because circulating free fatty acids (FFAs) are reported to inhibit IDE activity [29]; however, unfortunately we did not measure the blood level of FFAs. The effects of exercise on modulating amyloid precursor protein (APP) metabolism and the Aβ cascade are more probable mechanisms based on the report by Adlard et al., which demonstrated with their transgenic model mice for AD that one month of exercise decreased the proteolytic fragment of APP in the brain cortex independent of the mRNA or protein levels of IDE [30]. Of course, further investigations are needed into these issues, including the difference between the metabolism of two Aβ peptides, 40 and 42, and in their roles as biomarkers.

Our intervention resulted in a decrease not only in BMI but also in leg muscle mass in the ST group. The loss of leg muscle mass in spite of the training, the greater part of which comprised resistance training, may be due to the lack of instructions to the participants on energy intake during the intervention period, as well as the methodology of estimating muscle mass (impedance analysis). However, the muscle strength of the lower limbs was significantly improved by training in both groups. The findings are consistent with those by Moritani et al., which demonstrated that neural factors, not hypertrophy, absolutely contributed to muscle strength gain by resistance training in elderly people [31]. On the other hand, although the present study did not regard the risk factors of falling as the main outcome, our intervention, which included enough time for flexibility exercise, succeeded in improving only an aspect of motor ability, maximal step length. A re-examination of the exercise program is needed, so as to enhance balance and agility, two essential factors for elderly people in substantially reducing the risk of falling.

There are some limitations in the present study. First, the small number of participants may not be sufficient to verify the correlation between insulin resistance and metabolism of Aβ peptides. Second, the present study was conducted as single-blinded design, in which only the outcome assessors and the exercise instructor was aware of the character of each exercise program. However, it cannot be totally excluded that some participants had an idea of which training they were undergoing and that the issue influenced the greater improvement in cognitive function in the DT group. Third, because we did not treat the baseline BMI as a balancing factor at the randomization, the difference in the baseline BMI between the group, although it was not statistically significant, might also bring about study bias. Fourth, our 12-week intervention period was comparatively short, and a longer intervention is needed to elucidate the effect of dual-task training on preventing or slowing down cognitive decline. Finally, the validation of the Japanese version of the 3MS exam has not been thoroughly established yet because there are only a few previous reports using this device to evaluate cognitive functions [32].

## Conclusions

In conclusion, we demonstrated that cognitive-motor DT training was more beneficial than ST training alone in improving the broader domains of cognitive functions in elderly people and that the improvement was not directly due to the modulation of Aβ metabolism. Further studies are needed to address the utilities of the plasma levels of Aβ peptides as biomarkers of dementia, especially in Alzheimer’s disease, and the mechanisms concerning the improvement of cognitive functions by exercise.

## References

[1] Tinetti ME, Kumar C (2010). The patient who falls: “It’s always a trade-off”. J American Med Assoc.

[2] Lundin-Olsson L, Nyberg L, Gustafson Y (1997). “Stops walking when talking” as a predictor of falls in elderly people. Lancet.

[3] Yaffe K, Barnes D, Nevitt M, Lui LY, Covinsky K (2001). A prospective study of physical activity and cognitive decline in elderly women: women who walk. Arch Intern Med.

[4] Etgen T, Sander D, Huntgeburth U, Poppert H, Forstl H, Bickel H (2010). Physical activity and incident cognitive impairment in elderly persons: the INVADE study. Arch Intern Med.

[5] Lautenschlager NT, Cox KL, Flicker L, Foster JK, van Bockxmeer FM, Xiao J, Greenop KR, Almeida OP (2008). Effect of physical activity on cognitive function in older adults at risk for Alzheimer disease: a randomized trial. JAMA.

[6] Kemoun G, Thibaud M, Roumagne N, Carette P, Albinet C, Toussaint L, Paccalin M, Dugue B (2010). Effects of a physical training programme on cognitive function and walking efficiency in elderly persons with dementia. Dement Geriatr Cogn Disord.

[7] Jensen M, Schroder J, Blomberg M, Engvall B, Pantel J, Ida N, Basun H, Wahlund LO, Werle E, Jauss M (1999). Cerebrospinal fluid A beta42 is increased early in sporadic Alzheimer’s disease and declines with disease progression. Ann Neurol.

[8] Gravina SA, Ho L, Eckman CB, Long KE, Otvos L, Younkin LH, Suzuki N, Younkin SG (1995). Amyloid beta protein (A beta) in Alzheimer’s disease brain. Biochemical and immunocytochemical analysis with antibodies specific for forms ending at A beta 40 or A beta 42(43). J Biol Chem.

[9] Graff-Radford NR, Crook JE, Lucas J, Boeve BF, Knopman DS, Ivnik RJ, Smith GE, Younkin LH, Petersen RC, Younkin SG (2007). Association of low plasma Abeta42/Abeta40 ratios with increased imminent risk for mild cognitive impairment and Alzheimer disease. Arch Neurol.

[10] Malito E, Hulse RE, Tang WJ (2008). Amyloid beta-degrading cryptidases: insulin degrading enzyme, presequence peptidase, and neprilysin. Cellular Molecular Life Sci.

[11] Teng EL, Chui HC (1987). The Modified Mini-Mental State (3MS) examination. J Clin Psychiatry.

[12] Yokoyama H, Emoto M, Fujiwara S, Motoyama K, Morioka T, Komatsu M, Tahara H, Shoji T, Okuno Y, Nishizawa Y (2003). Quantitative insulin sensitivity check index and the reciprocal index of homeostasis model assessment in normal range weight and moderately obese type 2 diabetic patients. Diabetes Care.

[13] Matthews DR, Hosker JP, Rudenski AS, Naylor BA, Treacher DF, Turner RC (1985). Homeostasis model assessment: insulin resistance and beta-cell function from fasting plasma glucose and insulin concentrations in man. Diabetologia.

[14] Carvalho A, Rea IM, Parimon T, Cusack BJ (2014). Physical activity and cognitive function in individuals over 60 years of age: a systematic review. Clin Interv Aging.

[15] Erickson KI, Voss MW, Prakash RS, Basak C, Szabo A, Chaddock L, Kim JS, Heo S, Alves H, White SM (2011). Exercise training increases size of hippocampus and improves memory. Proc Natl Acad Sci U S A.

[16] Ruitenberg A, den Heijer T, Bakker SL, van Swieten JC, Koudstaal PJ, Hofman A, Breteler MM (2005). Cerebral hypoperfusion and clinical onset of dementia: the Rotterdam Study. Ann Neurol.

[17] Rhyu IJ, Bytheway JA, Kohler SJ, Lange H, Lee KJ, Boklewski J, McCormick K, Williams NI, Stanton GB, Greenough WT (2010). Effects of aerobic exercise training on cognitive function and cortical vascularity in monkeys. Neuroscience.

[18] Radak Z, Chung HY, Goto S (2005). Exercise and hormesis: oxidative stress-related adaptation for successful aging. Biogerontology.

[19] Stranahan AM, Zhou Y, Martin B, Maudsley S (2009). Pharmacomimetics of exercise: novel approaches for hippocampally-targeted neuroprotective agents. Curr Med Chem.

[20] Voss MW, Erickson KI, Prakash RS, Chaddock L, Malkowski E, Alves H, Kim JS, Morris KS, White SM, Wojcicki TR (2010). Functional connectivity: a source of variance in the association between cardiorespiratory fitness and cognition?. Neuropsychologia.

[21] Hawkins HL, Kramer AF, Capaldi D (1992). Aging, exercise, and attention. Psychol Aging.

[22] Smith PJ, Blumenthal JA, Hoffman BM, Cooper H, Strauman TA, Welsh-Bohmer K, Browndyke JN, Sherwood A (2010). Aerobic exercise and neurocognitive performance: a meta-analytic review of randomized controlled trials. Psychosom Med.

[23] Cooper C, Li R, Lyketsos C, Livingston G (2013). Treatment for mild cognitive impairment: systematic review. British J Psychiatry.

[24] Broadbent NJ, Squire LR, Clark RE (2004). Spatial memory, recognition memory, and the hippocampus. Proc Natl Acad Sci U S A.

[25] Rosano C, Venkatraman VK, Guralnik J, Newman AB, Glynn NW, Launer L, Taylor CA, Williamson J, Studenski S, Pahor M (2010). Psychomotor speed and functional brain MRI 2 years after completing a physical activity treatment. J Gerontol A Biol Sci Med Sci.

[26] de la Monte SM (2009). Insulin resistance and Alzheimer’s disease. BMB Rep.

[27] Karczewska-Kupczewska M, Lelental N, Adamska A, Nikolajuk A, Kowalska I, Gorska M, Zimmermann R, Kornhuber J, Straczkowski M, Lewczuk P (2013). The influence of insulin infusion on the metabolism of amyloid beta peptides in plasma. J Alzheimer’s Assoc.

[28] Baker LD, Frank LL, Foster-Schubert K, Green PS, Wilkinson CW, McTiernan A, Cholerton BA, Plymate SR, Fishel MA, Watson GS (2010). Aerobic exercise improves cognition for older adults with glucose intolerance, a risk factor for Alzheimer’s disease. J Alzheimer’s Dis.

[29] Hamel FG, Upward JL, Bennett RG (2003). In vitro inhibition of insulin-degrading enzyme by long-chain fatty acids and their coenzyme A thioesters. Endocrinology.

[30] Adlard PA, Perreau VM, Pop V, Cotman CW (2005). Voluntary exercise decreases amyloid load in a transgenic model of Alzheimer’s disease. J Neurosci.

[31] Moritani T, de Vries HA (1980). Potential for gross muscle hypertrophy in older men. J Gerontol.

[32] Yoshii Y, Tominaga D, Sugimoto K, Tsuchida Y, Hyodo A, Yonaha H, Kushi S (2008). Cognitive function of patients with brain tumor in pre- and postoperative stage. Surg Neurol.

